# *MET* alterations detected in blood-derived circulating tumor DNA correlate with bone metastases and poor prognosis

**DOI:** 10.1186/s13045-018-0610-8

**Published:** 2018-06-04

**Authors:** Sadakatsu Ikeda, Maria Schwaederle, Mandakini Mohindra, Denis L. Fontes Jardim, Razelle Kurzrock

**Affiliations:** 1 Department of Medicine, Center for Personalized Cancer Therapy, Division of Hematology/Oncology, University of California, San Diego, Moores Cancer Center, 3855 Health Sciences Drive, #0658, La Jolla, CA 92093-0987 USA; 2 Tokyo Medical and Dental University, Tokyo, Japan; 3 Department of Clinical Oncology, Hospital Sirio Libanes, Sao Paulo, Brazil

**Keywords:** MET, ctDNA, cfDNA, Liquid biopsies, Bone metastasis

## Abstract

**Background:**

We analyzed clinical associations of *MET* alterations in the plasma of patients with diverse malignancies.

**Methods:**

Digital sequencing of circulating tumor DNA (ctDNA) (54–70 genes) was performed in 438 patients; 263 patients also had tissue sequencing (182–315 genes). The most represented tumor types were gastrointestinal (28.1%), brain (24.9%), and lung (23.2%). Most patients (71.2%) had recurrent/metastatic disease.

**Results:**

*MET* alterations were observed in 31 patients (7.1%) and correlated with bone metastasis (*P* = 0.007), with *TP53* (*P* = 0.001) and *PTEN* (*P* = 0.003) abnormalities, and with an increased number of alterations (median, 4 vs 1, *P* = 0.001) (all multivariable analyses). Patients with *MET* alterations demonstrated a significantly shorter median time to metastasis/recurrence (1.0 vs 10.4 months, *P* = 0.044, multivariable) and a poorer survival (30.6 vs 58.4 months, *P* = 0.013, univariate only). Of the 31 patients with *MET* alterations, 18 also had tissue testing; only two also had tissue *MET* alterations (11.1%); *MET* alterations were detected at a lower frequency in tissue (1.14%) compared to ctDNA (7.1%), with *P* = 0.0002.

**Conclusions:**

In conclusion, the detection of *MET* alterations by liquid biopsy is feasible. *MET* ctDNA alterations were associated with a poorer prognosis, higher numbers of genomic abnormalities, and bone metastases. The correlation with bone metastases may explain the higher frequency of *MET* alterations in blood ctDNA than in tissue (since bones are rarely biopsied) and the previous observations of bone-predominant responses to MET inhibitors. The high number of co-altered genes suggests that MET inhibitors may need to be combined with other agents to induce/optimize responses.

**Electronic supplementary material:**

The online version of this article (10.1186/s13045-018-0610-8) contains supplementary material, which is available to authorized users.

## Background

MET, also called c-MET or hepatocyte growth factor (HGF) receptor, is a receptor tyrosine kinase discovered as an oncogene in the 1980s [1, 2]. Independent research found that HGF or scatter factor (SF) was a ligand for MET [3]. Upon binding of HGF to MET, the kinase domain phosphorylates growth factor receptor-bound protein 2 (GRB2) and GRB2-associated binding protein 1 (GAB1) and activates diverse downstream signaling pathways important in cancer, including the ERK/MAPK, PI3K-Akt/PKB, Crk-Rap, and Rac-Pak pathways [4]. These pathways form distinct branches that interact to regulate cell proliferation, invasion, migration, angiogenesis, development, organ regeneration, and tumorigenesis [4].

Activating *MET* mutations are found in diverse human cancer [5]. For instance, activating mutations in the kinase domain are a feature of both hereditary and some non-hereditary forms of papillary renal cell carcinoma [6]. *MET* amplification is seen in 5 to 20% of non-small cell lung cancer (NSCLC) and gastric cancer [7]. MET overexpression correlates with poor treatment outcome in some malignancies [8].

Drugs that target MET include inhibitors of the HGF/-MET pathway, MET antibodies, and MET kinase inhibitors [8]. Cabozantinib and crizotinib are both Food and Drug Administration (FDA)-approved multikinase inhibitors that are also potent suppressors of MET [9, 10]. Crizotinib induced responses in some patients with highly *MET-*amplified lung cancer [11]. Studies with the multi-target MET inhibitor cabozantinib have shown significant activity against a variety of solid tumors including melanoma, as well as renal and non-small cell lung, liver, medullary thyroid, breast, and ovarian cancer, but this activity is likely due to other targets of cabozantinib, such as VEGFR or RET [9]. In the phase I setting, patients with *MET* amplification did not respond to MET inhibitors (but the number of treated patients was small) [5].

Because of their non-invasive nature, liquid biopsies are increasingly used in the clinical setting. Indeed, numerous studies showed a relatively good correlation with tissue sequencing and the potential to detect actionable alterations [12–16]. In this study, we analyzed *MET* alterations in the plasma-derived circulating tumor DNA (ctDNA) of 438 patients with diverse malignancies and explored the relationship between *MET* alterations, demographics, as well as other molecular alterations and clinical outcomes.

## Methods

### Patients

We reviewed the clinicopathology and clinical outcomes of 438 consecutive patients with cancer for whom ctDNA testing had been performed and who were seen at the UC San Diego Moores Cancer Center from June 2014 to July 2016. Pathology was reviewed at UCSD. Data was abstracted from the electronic medical record. This study was performed and consents were obtained in accordance with the UCSD Institutional Review Board guidelines [PREDICT-UCSD (Profile Related Evidence Determining Individualized Cancer Therapy); NCT02478931].

### Next-generation sequencing

Digital sequencing of ctDNA (DNA) in all patients was performed by Guardant Health, Inc. (Guardant360, Redwood City, California, http://www.guardanthealth.com/guardant360/), a Clinical Laboratory Improvement Amendment (CLIA)-certified and College of American Pathologists (CAP)-accredited clinical laboratory. The analytical and clinical validation of Guardant360 was conducted in accordance with evidentiary standards established by the Standards for Reporting of Diagnostic Accuracy (STARD), REporting of tumor MARKer Studies (REMARK), Evaluation of Genomic Applications in Practice and Prevention (EGAPP), and the recent Next-generation Sequencing: Standardization of Clinical Testing (Nex-StoCT) biomarker guidelines [17]. As described in Lanman et al. [17], 5–30 ng of ctDNA was isolated from plasma (two 10 ml Streck tubes drawn for each patient) and sequencing libraries were prepared with custom in-line barcode molecular tagging and complete sequencing at 15,000× read depth (~ 4000 unique double-stranded cfDNA fragments, each represented by 3–5 sequencing reads). The panels utilize hybrid capture followed by NGS of the critical exons in a panel of 54–70 genes (Additional file 1: Table S1 to S3) and report all four major types of genomic alterations (point mutations, indels, fusions, and copy number amplifications). Post-sequencing bioinformatics matches the complementary strands of each barcoded DNA fragment to remove false positive results [17]. The variant allele fraction (VAF) is computed as the number of mutated DNA molecules divided by the total number (mutated plus wild type) of DNA fragments at that allele; VAF is reported as a percentage. The majority of cell-free DNA is wild type (germline); thus, the median VAF of somatic alterations is < 0.5%. The analytic sensitivity reaches detection of one to two single-mutant fragments from a 10-ml blood sample (0.1% limit of detection), and the analytic specificity is greater than 99.9999% [17].

For 144 patients, a 54-gene panel was used, which identified potential tumor-related alterations in 54 cancer-related genes (Additional file 1: Table S1) including copy number amplifications in *ERBB2*, *EGFR*, and *MET* (indels and fusions were not detected as part of this panel). For 272 patients, a 68-gene version of the original panel (expanded to all four major alteration types) was used, and for 22 patients, the most recent 70-gene panel version (further expanded to amplifications in 18 genes and fusions in 6 genes) was applied (Additional file 1: Table S2 and S3). Only non-synonymous alterations were included in our analysis.

In addition, 263 patients (~ 60%) of the 438 patients with ctDNA test also had CLIA/CAP-accredited next-generation sequencing (NGS) performed on tumor tissue (FoundationOne™, Cambridge, Massachusetts, http://www.foundationone.com.) (*N* = 182 to 315 gene panels).

### Statistical and outcome analysis

Patient characteristics were summarized using descriptive statistics. Medians and respective 95% confidence intervals and range were calculated, whenever possible. Associations between categorical variables were tested using a binary logistic regression model. Linear variables were tested using the Mann-Whitney *U* test for univariable analyses and a multiple linear regression model for multivariable analyses.

Time to metastasis/recurrence was defined as the time interval between diagnosis and first metastasis/recurrence (whichever came first) or last follow-up date (patients who had not recurred/developed metastases at last follow-up were censored on that date). Overall survival (OS) was defined as the time from diagnosis to death or last follow-up date for patients who were alive (patients still alive at the last follow-up were censored on that date). Estimations for the time to first metastasis/recurrence and OS were done using Kaplan-Meier analyses and were compared among subgroups by the log-rank test for univariable analysis or Cox regression models for multivariable analysis. All statistical analyses were performed by author MS with SPSS version 24.0.

## Results

### Patient demographic characteristics

The median age of patients at diagnosis was 57.5 years (CI 95%, 54.5–59.1). Women comprised 52.1% (*N* = 228) of the population. The majority of patients were Caucasian (69.2%, *N* = 303). The most represented tumor types were gastrointestinal (28.1%, *N* = 123), brain (24.9%, *N* = 109), lung (23.2%, *N* = 102), and breast (11.6%, *N* = 51) cancers. The majority of the patients had recurrent or metastatic disease at the time of blood draw used for testing (71.2%, *N* = 312) **(**Table 1).

**Table 1:** Demographics comparison of 438 patients with or without *MET* alterations

Characteristics	Total patients, *N* = 438 (100%)	*MET* wild type, *N* = 407 (92.9%)	*MET* alteration(s), *N* = 31 (7.1%)	*P* values*
Age at diagnosis (years) (median, CI 95%)	57.5 (54.5–59.1)	57.7 (55.3–59.2)	53.8 (49.0–62.2)	0.791
Gender				0.288
Women	228 (52.1%)	209 (91.7%)	19 (8.3%)	
Men	210 (47.9%)	198 (94.3%)	12 (5.7%)	
Ethnicity				
Caucasian	303 (69.2%)	284 (93.7%)	19 (6.3%)	0.326
Asian	52 (11.9%)	46 (88.5%)	6 (11.5%)	0.188
Hispanic	30 (6.8%)	28 (93.3%)	2 (6.7%)	0.928
African American	9 (2.1%)	8 (88.9%)	1 (11.1%)	0.637
Middle Eastern	5 (1.1%)	5 (100%)	0 (0%)	0.998
Unknown	39 (8.9%)	36 (92.3%)	3 (7.7%)	0.875
Type of cancer				
Gastrointestinal	123 (28.1%)	118 (95.9%)	5 (4.1%)	0.132
Brain	109 (24.9%)	107 (98.2%)	2 (1.8%)	0.026**
Lung	102 (23.2%)	90 (88.2%)	12 (11.8%)	0.039**
Breast	51 (11.6%)	45 (88.2%)	6 (11.8%)	0.172
Genitourinary	18 (4.1%)	15 (83.3%)	3 (16.7%)	0.120
Head and neck	10 (2.3%)	9 (90.0%)	1 (10.0%)	0.717
Gynecologic	7 (1.6%)	6 (85.7%)	1 (14.3%)	0.465
Melanoma	5 (1.1%)	4 (80.0%)	1 (20.0%)	0.285
Hematologic	3 (0.7%)	3 (100%)	0 (0%)	0.998
Other^a^	10 (2.3%)	10 (100%)	0 (0%)	0.998
Presence of metastasis or recurrence at the time of blood draw				
Yes	312 (71.2%)	284 (91.0%)	28 (9.0%)	0.024**
No	126 (28.8%)	123 (97.6%)	3 (2.4%)	

The percentages on the first column are expressed over the total number of patients (*N* = 438); for the second and third columns, percentages are expressed over the total number of patients for each variable

^a^Lymphoma (*n* = 2), sarcoma (*n* = 2), thymoma (*n* = 2), desmoid tumor, neurofibromatosis, peripheral nerve sheath tumor, and carcinoma of unknown primary (each *n* = 1)

*P values were computed using the independent sample Mann-Whitney *U* test for linear variables (age at diagnostic) and the logistic binary regression analysis for categorical variables, as appropriate

**MET alterations were negatively associated with brain tumors but positively associated with lung tumors and metastasis or recurrence at the time of blood draw

### *MET* alterations and associations with patient characteristics

**Fig. 1:**
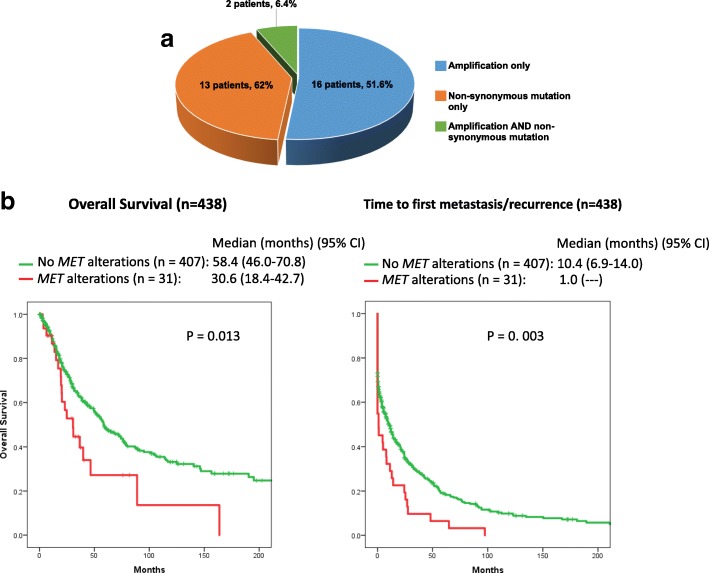
**a** *MET* alterations representation. Pie chart representing the different types of MET alterations identified in our cohort comprising 438 patients. **b** Overall survival and time to first metastasis/recurrence. Kaplan-Meier curves depicting the overall survival (left panel) and the time to first metastasis/recurrence (both from diagnosis time). *P* values are from univariable analysis. For more details, refer to Tables 4 and 5

Overall, *MET* alterations were observed in 31 of the 438 patients whose ctDNA was tested (7.1%). Sixteen patients had a *MET* amplification only; 13 had a somatic mutation only; and two had both an amplification and a somatic *MET* mutation (Fig. 1a).

#### Univariate analyses

*MET* alterations were significantly (positively) associated with lung cancers (11.8%) and the presence of metastasis/recurrence at the time of blood draw and negatively associated with brain tumors 1.8% (all *P* < 0.05) (Table 1). We then examined the associations with other genomic alterations and found that *MET* alterations correlated in univariable analysis with alterations in *TP53*, *EGFR*, *PIK3CA*, *BRAF*, *ARID1A*, *ALK*, and *PTEN* genes (all *P* < 0.05, Additional file 1: Table S4). We also investigated the potential associations between *MET* alterations and the location of metastatic sites and showed a significant correlation with lymph nodes and bone metastasis (both *P* < 0.02, Additional file 1: Table S4). Of note, patients with *MET* alterations had a median of two metastatic sites versus a single site for patients without *MET* alterations (*P* = 0.001). The latter conclusions remained unchanged if the patients with brain tumors, lymphoma/leukemia, thymoma, and NF were excluded, with *P* values of < 0.05 for association between *MET* alterations and lymph node or bone metastases and increased number of metastases.

#### Multivariate analyses

In the multiple logistic regression model (that included any parameters that were significant (*P* ≤ 0.05) in univariate analysis), the only variables that remained statistically associated with *MET* alterations were aberrations in *TP53* (*P* = 0.001) and *PTEN* (*P* = 0.003) genes, as well as an increased incidence of bone metastasis (*P* = 0.007) (Table 2). The univariate association between *MET* alterations and lung cancer, the negative association with brain tumors, and the correlation with metastases at the time of blood draw did not remain significant in multivariate analysis. Multivariable analysis also demonstrated that *MET* alterations correlated with an increased number of alterations (median of 4 alterations vs 1, *P* = 0.001) (Table 3). These conclusions remained valid if the patients with brain tumors, lymphoma/leukemia, thymoma, and NF were excluded, with *P* values of < 0.01 for association between *MET* alterations and *TP53* or *PTEN* alterations, bone metastases, and increased number of metastases.

**Table 2:** Multivariable analysis of the variables associated with *MET* alterations*

	*MET* wild type, *N* = 407	*MET* altered, *N* = 31	Univariable			Multivariable		
Characteristics			Wald	Odds ratio (CI 95%)	*P* value	Wald	Odds ratio (CI 95%)	*P* value
Type of cancer								
Brain (*n* = 109)**	107 (26.3%)	2 (6.5%)	4.94	0.19 (0.05–0.83)	0.026**	–	–	–
Lung (*n* = 102)	90 (22.1%)	12 (38.7%)	4.26	2.22 (1.04–4.76)	0.039	–	–	–
Presence of metastasis or recurrence at the time of blood draw (*n* = 312)	284 (69.8%)	28 (90.3%)	5.13	4.0 (1.2–14.3)	0.024	–	–	–
Genetic alteration type								
*TP53* (*n* = 149)	129 (31.7%)	20 (64.5%)	12.2	3.85 (1.82–8.33)	< 0.001	10.9	3.7 (1.7–8.3)	*0.001*
*EGFR* (*n* = 53)	43 (10.6%)	10 (32.4%)	11.2	4.0 (1.79–9.1)	0.001	–	–	–
*PIK3CA* (*n* = 42)	35 (8.6%)	7 (22.6%)	5.93	3.1 (1.25–7.7)	0.015	–	–	–
*BRAF* (*n* = 27)	22 (5.4%)	5 (16.1%)	5.14	3.3 (1.2–10.0)	0.023	–	–	–
*ARID1A* (*n* = 19)	15 (3.7%)	4 (12.9%)	5.14	3.85 (1.2–12.5)	0.023	–	–	–
*ALK* (*n* = 14)	11 (2.7%)	3 (9.7%)	3.94	3.85 (1.02–14.3)	0.047	–	–	–
*PTEN* (*n* = 9)	6 (1.5%)	3 (9.7%)	7.72	7.1 (1.7–33.3)	0.007	9.12	11.1 (2.3–50.0)	*0.003*
Metastatic sites								
Lymph node (*n* = 139)	123 (30.2%)	16 (51.6%)	5.77	2.4 (1.2–5.3)	0.016	–	–	–
Bone (*n* = 102)	88 (21.6%)	14 (45.2%)	8.26	2.9 (1.4–6.3)	0.004	7.34	2.9 (1.35–6.25)	*0.007*
No metastases (*n* = 147)**	145 (35.6%)	2 (6.5%)	7.96	0.13 (0.03–0.5)	0.005**	–	–	–

The Wald statistics test the unique contribution of each variable; the higher the Wald statistics, the higher the association/contribution in the model

*Only variables that were significant in the univariable models (logistic regression) were included in the multivariable analysis, with the final model containing only significant covariates in the multivariable analyses (forward stepwise selection model)

**“Brain tumors” and “no metastases” were negatively associated with *MET* alterations in univariate analysis. These variables were not significant in the final multivariate analysis model

**Table 3:** Association with the number of alterations*

Variables	Median *N* of alterations (95% CI) (range)	*P* value (univariable)	*t* statistic (multivariable)	*P* value (multivariable)
Overall	1 (1–1) (0–26)			
Tumor type				
Gastrointestinal	2 (1–2) (0–26)	0.031	–	–
Brain	0 (0–0) (0–5)	< 0.001	–	–
Lung	2 (2–3) (0–21)	< 0.001	–	–
Genomic alterations				
*MET*	4 (3–6) (1–21)	< 0.001	3.35	*0.001*
*TP53*	3 (3–3) (1–21)	< 0.001	6.86	*< 0.001*
*EGFR*	3 (3–4) (1–26)	< 0.001	–	–
*PIK3CA*	5 (4–6) (1–26)	< 0.001	7.31	*< 0.001*
*BRAF*	5 (4–6) (1–26)	< 0.001	6.54	*< 0.001*
*KRAS*	4 (3–4) (1–26)	< 0.001	6.01	*< 0.001*
*MYC*	5 (3–6) (2–7)	< 0.001	2.70	*0.007*
Metastatic/recurrence at the time of blood draw	2 (1–2) (0–26)	< 0.001	–	–
Metastatic sites				
Lymph node	2 (2–3) (0–21)	< 0.001	2.05	*0.041*
Bone	2 (2–3) (0–26)	< 0.001	–	–
Liver	2 (2–3) (0–26)	< 0.001	–	–
Lung	2 (1–3) (0–19)	0.010	–	–
Brain	2 (1–3) (0–21)	< 0.001	–	–
Adrenal	2 (2–4) (0–20)	< 0.001	2.06	*0.040*

*Only variables with ≥ 20 patients in the overall population were tested. Only significant variables in the univariable analysis (non-parametric Mann-Whitney *U* test) were included in the multivariable model (multiple linear regression), with the final model containing only significant covariates (stepwise model selection). The *t* statistics test the unique contribution of each variable; the higher the *t* statistics, the higher the association/contribution in the model

The other variables that were significantly associated with a higher number of alterations in the multivariable analysis were alterations in *TP53*, *PIK3CA*, *BRAF*, *KRAS*, or *MYC* genes as well as the presence of metastasis in the lymph node or adrenal tissue (Table 3).

### *MET* alterations and survival outcomes

#### Overall survival

Overall, 213 patients (213/438, 48.6%) had died at the time of our analysis (20/31 with *MET* alterations; 193/407 without *MET* alterations detected in their ctDNA). A log rank test demonstrated a poorer survival (time from diagnosis until death or last follow-up date) for patients bearing *MET* alterations (30.6 months vs 58.4, *P* = 0.013). In the multivariable analysis, only lung cancer as well as the presence of *ARID1A*, *KRAS*, *ALK*, and *MYC* alterations and liver metastasis remained significant predictors of a poorer survival (all *P* < 0.50, Fig. 1b) (Table 4).

**Table 4:** Overall survival analysis (*N* = 438 patients)*

	Univariable		Multivariable		
Characteristics	Median time (months) (std. error)	*P* value	Wald	Hazard ratio (CI 95%)	*P* value
Type of cancer					
Gastrointestinal (*n* = 123) vs not	36.3 (4.5) vs 71.6 (6.5)	0.002	–	–	–
Brain (*n* = 109) vs not**	95.0 (50.1) vs 51.5 (5.9)	0.003	–	–	–
Lung (*n* = 102) vs not	31.8 (6.0) vs 61.0 (7.4)	0.002	9.59	1.7 (1.21–4.04)	*0.002*
Breast (*n* = 51) vs not	104.8 (20.3) vs 50.0 (5.6)	0.027			
Genetic alteration type					
*MET* (*n* = 31) vs not	30.6 (6.2) vs 58.4 (6.3)	0.013	–	–	–
*TP53* (*n* = 149) vs not	42.1 (6.1) vs 66.5 (10.3)	0.003	–	–	–
*PIK3CA* (*n* = 42) vs not	43.2 (10.6) vs (58.4 (6.3)	0.013	–	–	–
*ARID1A* (*n* = 19) vs not	24.8 (6.2) vs 58.4 (6.8)	0.002	7.98	2.7 (1.25–4.0)	*0.005*
*KRAS* (*n* = 50) vs not	18.3 (7.3) vs 59.7 (6.5)	< 0.001	15.68	2.1 (1.5–3.12)	*< 0.001*
*ALK* (*n* = 14) vs not	31.3 (9.0) vs 58.3 (6.7)	0.015	5.76	2.04 (1.14–3.7)	*0.016*
*SMAD4* (*n* = 12) vs not	21.7 (8.2) vs 58.3 (6.2)	0.007	–	–	–
*MYC* (*n* = 20) vs not	22.2 (3.9) vs 59.1 (6.5)	< 0.001	7.59	2.17 (1.24–3.77)	*0.006*
Metastatic/recurrence at the time of blood draw vs not	53.6 (4.9) vs NR	0.011	–	–	–
Metastatic sites					
Liver (*n* = 111) vs not	49.3 (8.2) vs 59.1 (7.9)	0.023	4.85	1.4 (1.03–1.89)	*0.028*
Lymph node (*n* = 139) vs not	39.9 (7.4) vs 74.1 (11.1)	< 0.001	–	–	–

Only variables that were significant in the univariable models (log rank test) were included in the multivariable analysis (Cox regression model), with the final model containing only significant covariates in the multivariable analyses (forward stepwise selection model). The Wald statistics test the unique contribution of each variable; the higher the Wald statistics, the higher the association/contribution in the model

NR not reached

*Overall survival was defined as the time from diagnosis to death or last follow-up date

**Brain tumors (*N* = 109) included *n* = 50 glioblastoma cases, while the rest of the tumors were lower grade astrocytomas or other lower grade brain tumors

#### Time to metastasis/recurrence

Patients with *MET* alterations had a significantly shorter median time to metastasis/recurrence, with a median of 1.0 months (95%CI could not be computed) versus 10.4 months (95%CI 6.9–14.0) (*P* = 0.003) (Table 5). *MET* alterations remained significantly associated with a shorter time to metastasis/recurrence in a multivariable analysis (*P* = 0.044), along with the presence of liver (*P* = 0.022) or lymph node metastases (*P* < 0.001). Inversely, breast cancer correlated with a longer time to metastasis/recurrence (*P* < 0.001) (Fig. 1b and Table 5).

**Table 5:** Time to metastasis/recurrence from diagnosis (*N* = 438 patients)*

	Univariable		Multivariable		
Characteristics	Median time (months) (95% CI)	*P* value	Wald	Hazard ratio (CI 95%)	*P* value
Type of cancer					
Gastrointestinal (*n* = 123) vs not	0.67 (0–1.9) vs 14.1 (8.3–19.8)	< 0.001	–	–	–
Brain (*n* = 109) vs not	42.8 (19.0–66.7) vs 2.5 (0.49–4.4)	< 0.001	–	–	–
Lung (*n* = 102) vs not	0.6 (0–1.3) vs 14.0 (9.5–18.6)	< 0.001	–	–	–
Breast (*n* = 51) vs not	30.4 (14.3–46.4) vs 7.0 (3.8–10.2)	0.001	40.03	0.33 (0.23–0.47)	*< 0.001*
Genitourinary (*n* = 18) vs not	0 (−) vs 10.1 (6.8–13.4)	0.020	–	–	–
Head and neck (*n* = 10) vs not	0 (−) vs 9.8 (6.2–13.4)	0.015	–	–	–
Genetic alteration type					
*MET* (*n* = 31) vs not	1.0 (−) vs 10.4 (6.9–14.0)	0.003	4.05	1.47 (1.01–2.13)	*0.044*
*TP53* (*n* = 149) vs not	4.1 (0.5–7.7) vs 12.7 (6.1–19.3)	0.001	–	–	–
*EGFR* (*n* = 53) vs not	3.6 (0–8.2) vs 11.4 (7.9–14.8)	0.003	–	–	–
*ARID1A* (*n* = 19) vs not	3.6 (0–10.1) vs 10.0 (6.3–13.7)	0.013	–	–	–
*KRAS* (*n* = 50) vs not	0 (−) vs 11.4 (8.3–13.7)	< 0.001	–	–	–
*SMAD4* (*n* = 12) vs not	0 (−) vs 10.1 (6.7–13.4)	0.040	–	–	–
*MYC* (*n* = 20) vs not	0.2 (0–0.75) vs 10.4 (7.0–13.9)	< 0.001	–	–	–
Metastatic sites					
Adrenal (*n* = 29) vs not	0 (−--) vs 11.3 (8.0–14.6)	0.002	–	–	–
Bone (*n* = 102) vs not	1.5 (0–6.7) vs 11.4 (8.2–14.5)	0.049	–	–	–
Liver (*n* = 111) vs not	0.9 (0–2.6) vs 132.3 (9.7–16.9)	< 0.001	10.04	1.49 (1.16–1.92)	*0.022*
Lymph node (*n* = 139) vs not	0.3 (−) vs 20.1 (14.3–25.9)	< 0.001	13.41	1.54 (1.22–1.92)	*< 0.001*
Peritoneal (*n* = 49) vs not	0 (−) vs 11.3 (8.1–14.5)	< 0.001	–	–	–
Brain (*n* = 63) vs not	3.6 (0–7.8) vs 10.4 (7.0–13.9)	0.045	–	–	–
Lung (*n* = 90) vs not	1.2 (0–8.0) vs 11.3 (7.7–15.0)	0.003	–	–	–

The Wald statistics test the unique contribution of each variable; the higher the Wald statistics, the higher the association/contribution in the model

*Only variables that were significant in the univariable models (log rank test) were included in the multivariable analysis (Cox regression model), with the final model containing only significant covariates in the multivariable analyses (forward stepwise selection model). For some values, the 95% CI could not be computed (−)

### Comparison with tissue testing

As noted, 438 patients had ctDNA testing; 263 of these patients also had tissue NGS performed.

Of the 31 patients with *MET* alterations in ctDNA, 18 also had tissue testing (Foundation Medicine see the “Methods” section). The median time interval between the blood draw and the tissue biopsy for these 18 patients was 6.1 months (95% CI (2–13.7); range (0.2–32.6)). In most of the patients with both types of testing, the ctDNA test was performed after the tissue testing (16/18 cases). Only two patients who had a *MET* alteration identified in their ctDNA also had a *MET* alteration found in their tissue testing (11.1%; *MET* amplification and *MET* Y501C, one patient each). In these two patients, the time interval between the ctDNA and tissue biopsies was 1.8 and 15.3 months, and both tissues used for the testing were from the primary tumor.

In only one patient, a *MET* amplification was detected in the tissue and not in ctDNA (1 of 263 total patients who had NGS tissue testing). The tissue test was performed on a pancreatic tumor that was surgically removed, and the ctDNA test was done more than 1 year later (recent scans showed appearance of new pulmonary and liver nodules 2 months prior the ctDNA testing).

Overall, MET alterations were detected at a significantly lower frequency in tissue (3/263 patients, 1.14%) compared to ctDNA (31/438, 7.1%), *P* = 0.0002. Further, of eight patients who harbored *MET* alterations in ctDNA, and had ctDNA and tissue testing within 2 months of each other, only one patient showed a similar MET alteration in the tissue. Six of the seven patients with only ctDNA positive for *MET* alterations had bone metastases.

## Discussion

This is the largest study interrogating the feasibility and utility of *MET* alteration detection through blood-derived ctDNA. Liquid biopsy is a non-invasive method to find genomic aberrations and is increasingly utilized in the clinical setting as reflected by the non-small cell lung cancer National Comprehensive Cancer Network (NCCN) guidelines [18, 19]. Our study demonstrated that *MET* ctDNA alterations were detected in 7.1% of patients with solid tumors. This detection rate is higher compared to previous tissue studies [5, 20, 21]. For instance, a study of *MET* tissue amplification determined by fluorescent in situ hybridization (FISH) demonstrated that 2.6% of 1115 solid tumor specimens were positive [5]. Furthermore, the MSKCC-IMPACT study showed that 3% of patients had *MET* tissue alterations [20, 21]. The differences in rate of *MET* alterations between our study and the other studies could be due to following reasons: (i) our technology detected both single nucleotide substitutions and amplifications, while the previous investigation by Jardim et al. at MD Anderson Cancer Center [5] discerned only amplifications (though the MSKCC study would have discerned both single nucleotide substitutions and amplifications [21]); (ii) our study used blood-derived ctDNA, which could capture shed tumor DNA from multiple sites, while the previous reports used tissue-based testing, which would only detect aberrations in the piece of tissue biopsied. Consistent with the above observations, our tissue NGS testing also showed significantly lower rates of *MET* alterations than the ctDNA NGS: 3 of 263 patients (1.1%) (who also had ctDNA tests) were positive for *MET* alterations in tissue versus 31 of 438 ctDNA-tested patients (7%) being positive for *MET* alterations (*P* = 0.0002). Further, of 18 patients positive for *MET* ctDNA alterations who also had tissue NGS, only 2 (11.1%) were also positive for a *MET* alteration by tissue NGS. The biologic underpinnings of discordance between ctDNA and tissue NGS results have been previously documented and include spatial (intra-tumor and inter-tumor) and temporal heterogeneity in genomic anomalies in cancers along with the fact that ctDNA is comprised of DNA that has leaked into the circulation from diverse metastatic sites while tissue NGS reflects only the tissue specimen analyzed [14].

The question that arises is whether or not biological explanations can specifically account for the higher rate of *MET* alterations in ctDNA. Of interest in this regard, our study showed that bone metastases were independently correlated with *MET* alterations (Additional file 1: Table S2 and Table S4). Indeed, 14 of 31 patients (45%) positive for *MET* ctDNA alterations had bone metastases (Additional file 1: Table S4). Of possible relevance in this regard**,** MET inhibitors are known to show efficacy in bone lesions [22, 23]. In the COMET-1 trial, 682 patients with castrate-resistant prostate cancer who progressed after docetaxel and androgen modulators (abiraterone and/or enzalutamide) were randomly assigned to either cabozantinib (MET inhibitor) or prednisone. Although there was no difference in overall survival, 42% of cabozantinib-treated patients showed bone scan response compared to 3% of prednisone-treated patients (*P* < 0.001), albeit without prostate surface antigen (PSA) response [24]. In the METEOR trial, 658 patients with advanced renal cell carcinoma who progressed with at least one VEGFR small molecule inhibitor were randomized to the MET inhibitor cabozantinib or the mTOR inhibitor everolimus [25]. In a sub-group analysis, patients randomized to cabozantinib arm with bone metastasis were associated with better overall survival (OS) (hazard ratio (HR) 0.54, 95% confidence interval (CI) 0.34–0.84) compared with non-bone metastasis (HR 0.71, 95% CI 0.55–0.91) [25]. These data together with our results raise the possibility that cancer clones with *MET* alterations preferentially localize to bone and may therefore explain bone responses after MET inhibitor therapy. Tissue biopsies are rarely performed on bones (none of our 18 patients with *MET* alterations in ctDNA who also had tissue NGS had a bone biopsy). Of interest in this regard, MET is prominently expressed (as determined by immunohistochemistry) at the site of bone metastases in renal cell cancer [26]. It is therefore conceivable that the high rates of ctDNA positivity for *ME*T alterations, which strongly and independently correlated with bone metastases in our study, reflect shed *MET* alteration-bearing DNA from bone lesions (Additional file 1: Table S2 and Table S4).

Our study also found that *MET* ctDNA alterations are associated with poor prognosis, including decreased survival and shorter time to recurrence/metastasis (Tables 4 and 5, Fig. 1b). These results are consistent with those found by correlating *MET* alterations found in tissue NGS with outcome in specific malignancies, such as astrocytomas [27], breast cancers [28], genitourinary malignancies [29], and ovarian [30] or gastric/esophageal cancers [31]. Specific genes that were co-altered with *MET* in multivariate analysis of our study participants include *PTEN* and *TP53* (Table 2); previously, another study has also shown the association between *MET* and *PTEN* abnormalities [5]. Overall, *MET* alterations significantly correlated with a higher number of alterations, which may explain—at least in part—the relatively limited efficacy of MET inhibitors as single agents for the treatment of *MET*-altered advanced malignancies observed in prior studies [5, 29–31]. Indeed, if patients with *MET* alterations generally have multiple genomic abnormalities, it is likely that combination therapy, rather than monotherapy with a MET inhibitor, may be necessary to achieve salutary effects.

Our study has several limitations. First, only 60% of our patients (*N* = 263) with ctDNA analyses also had tissue sequencing and the median time between the two tests in the patients with *MET-*altered ctDNA was about 6 months. Second, our patients did not have bone biopsies done, which would be of interest to determine if *MET* alterations are found in such biopsies, since ctDNA *MET* alterations correlate independently with the presence of bone metastases. Third, though our study with 438 patients is the largest to date, the rarity of *MET* alterations suggests that investigations of even greater numbers of patients may be worthwhile in order to best understand the biology and correlations of *MET* alterations.

## Conclusions

In summary, our study demonstrated that assessment of *MET* genomic aberrations by liquid biopsy is feasible. We found that *MET* ctDNA anomalies were associated with bone metastases, multiple genomic alterations, and a poorer prognosis, including poorer overall survival and a shorter time to recurrence/metastases. Further studies are needed to better understand the biologic relationship between *MET* alterations and bone lesions, and next-generation trials with MET inhibitors may require combinations of drugs that address the genes such as *PTEN* that are frequently co-altered in these patients.

## Additional file

Additional file 1: **Table S1.** 54-gene panel (*N* = 122 patients)— identifies potential tumor-related genomic alterations within 54 cancer-related genes including amplifications in *ERBB2*, *EGFR*, and *MET*. Only non-synonymous alterations were analyzed. **Table S2.** 68-gene panel (*N* = 272 patients), comprising amplifications in 16 genes as well as some fusions and indels. Only non-synonymous alterations were analyzed. **Table S3.** 70-gene panel (*N* = 22 patients). Only non-synonymous alterations were analyzed. **Table S4.** Comparison of clinical characteristics in 438 patients with or without *MET* alterations (univariate analysis). (DOCX 20 kb)

## Abbreviations

95% CI: 95% confidence interval
CAP: College of American Pathologists
ctDNA: Circulating tumor DNA
FDA: Food and Drug Administration
FISH: Fluorescent in situ hybridization
GAB1: GRB2-associated binding protein 1
GRB2: Growth factor receptor-bound protein 2
HGF: Hepatocyte growth factor
HR: Hazard ratio
NCCN: National Comprehensive Cancer Network
NGS: Next-generation sequencing
NSCLC: Non-small cell lung cancer
OS: Overall survival
PREDICT: Profile Related Evidence Determining Individualized Cancer Therapy
VAF: Variant allele fraction

## References

[1] Cooper CS, Park M, Blair DG, Tainsky MA, Huebner K, Croce CM (1984). Molecular cloning of a new transforming gene from a chemically transformed human cell line. Nature.

[2] Park M, Dean M, Kaul K, Braun MJ, Gonda MA, Vande WG (1987). Sequence of MET protooncogene cDNA has features characteristic of the tyrosine kinase family of growth-factor receptors. Proc Natl Acad Sci U S A.

[3] Bottaro DP, Rubin JS, Faletto DL, Chan AM, Kmiecik TE, Vande Woude GF (1991). Identification of the hepatocyte growth factor receptor as the c-met proto-oncogene product. Science.

[4] Birchmeier C, Birchmeier W, Gherardi E, Vande Woude GF (2003). Met, metastasis, motility and more. Nat Rev Mol Cell Biol.

[5] Jardim DLF, Tang C, Gagliato DDM, Falchook GS, Hess K, Janku F (2014). Analysis of 1,115 patients tested for MET amplification and therapy response in the MD Anderson Phase I Clinic. Clin Cancer Res.

[6] Schmidt L, Duh FM, Chen F, Kishida T, Glenn G, Choyke P (1997). Germline and somatic mutations in the tyrosine kinase domain of the MET proto-oncogene in papillary renal carcinomas. Nat Genet.

[7] Kawakami H, Okamoto I, Okamoto W, Tanizaki J, Nakagawa K, Nishio K (2014). Targeting MET amplification as a new oncogenic driver. Cancers.

[8] Madoz-Gúrpide J, Zazo S, Chamizo C, Casado V, Caramés C, Gavín E (2015). Activation of MET pathway predicts poor outcome to cetuximab in patients with recurrent or metastatic head and neck cancer. J Transl Med.

[9] Kurzrock R, Sherman SI, Ball DW, Forastiere AA, Cohen RB, Mehra R (2011). Activity of XL184 (cabozantinib), an oral tyrosine kinase inhibitor, in patients with medullary thyroid cancer. J Clin Oncol.

[10] Shaw AT, Kim DW, Nakagawa K, Seto T, Crinó L, Ahn M-J (2013). Crizotinib versus chemotherapy in advanced ALK-positive lung cancer. N Engl J Med.

[11] Ross C, Sai-Hong IO, Shapiro G (2014). Efficacy and safety of crizotinib in patients with advanced c MET-amplified non-small cell lung cancer (NSCLC). JCO.

[12] Schwaederle M, Patel SP, Husain H, Ikeda M, Lanman R, Banks KC (2017). Utility of genomic assessment of blood-derived circulating tumor DNA (ctDNA) in patients with advanced lung adenocarcinoma. Clin Cancer Res.

[13] Kato S, Krishnamurthy N, Banks KC, De P, Williams K, Williams C (2017). Utility of genomic analysis in circulating tumor DNA from patients with carcinoma of unknown primary. Cancer Res.

[14] Schwaederle M, Husain H, Fanta PT, Piccioni DE, Kesari S, Schwab RB (2016). Use of liquid biopsies in clinical oncology: pilot experience in patients. Clin Cancer Res.

[15] Bidard F-C, Madic J, Mariani P, Piperno-Neumann S, Rampanou A, Servois V (2014). Detection rate and prognostic value of circulating tumor cells and circulating tumor DNA in metastatic uveal melanoma. Int J Cancer.

[16] Schwaederle M, Husain H, Fanta PT, Piccioni DE, Kesari S, Schwab RB (2016). Detection rate of actionable mutations in diverse cancers using a biopsy-free (blood) circulating tumor cell DNA assay. Oncotarget.

[17] Lanman RB, Mortimer SA, Zill OA, Sebisanovic D, Lopez R, Blau S (2015). Analytical and clinical validation of a digital sequencing panel for quantitative, highly accurate evaluation of cell-free circulating tumor DNA. PLoS One.

[18] Kwapisz D (2017). The first liquid biopsy test approved. Is it a new era of mutation testing for non small cell lung cancer?. Ann Transl.

[19] Chabon JJ, Simmons AD, Lovejoy AF, Esfahani MS, Newman AM, Haringsma HJ (2016). Circulating tumour DNA profiling reveals heterogeneity of EGFR inhibitor resistance mechanisms in lung cancer patients. Nat Commun.

[20] Cheng DT, Mitchell TN, Zehir A, Shah RH, Benayed R, Syed A (2015). Memorial Sloan Kettering-integrated mutation profiling of actionable cancer targets (MSK-IMPACT): a hybridization capture-based next-generation sequencing clinical assay for solid tumor molecular oncology. J Mol Diagn.

[21] Zehir A, Benayed R, Shah RH, Syed A, Middha S, Kim HR (2017). Mutational landscape of metastatic cancer revealed from prospective clinical sequencing of 10,000 patients. Nat Med.

[22] Graham TJ, Box G, Tunariu N, Crespo M, Spinks TJ, Miranda S (2014). Preclinical evaluation of imaging biomarkers for prostate cancer bone metastasis and response to cabozantinib. J Natl Cancer Inst.

[23] McKay RR, Kroeger N, Xie W, Lee J-L, Knox JJ, Bjarnason GA (2014). Impact of bone and liver metastases on patients with renal cell carcinoma treated with targeted therapy. Eur Urol.

[24] Smith M, De Bono J, Sternberg C, Le Moulec S, Oudard S, De Giorgi U (2016). Phase III study of cabozantinib in previously treated metastatic castration-resistant prostate cancer: COMET 1. J Clin Oncol.

[25] Choueiri TK, Escudier B, Powles T, Tannir NM, Mainwaring PN, Rini BI (2016). Cabozantinib versus everolimus in advanced renal cell carcinoma (METEOR): final results from a randomised, open-label, phase 3 trial. Lancet Oncol.

[26] Mukai S, Yorita K, Kawagoe Y, Katayama Y, Nakahara K, Kamibeppu T (2015). Matriptase and MET are prominently expressed at the site of bone metastasis in renal cell carcinoma: immunohistochemical analysis. Hum Cell.

[27] Pierscianek D, Kim YH, Motomura K, Mittelbronn M, Paulus W, Brokinkel B (2013). MET gain in diffuse astrocytomas is associated with poorer outcome. Brain Pathol.

[28] de Melo Gagliato D, Fontes Jardim DL, Falchook G, Tang C, Zinner R (2014). Analysis of MET genetic aberrations in patients with breast cancer at MD Anderson Phase I Unit. Clin Breast Cancer.

[29] Jardim DLF, de Melo Gagliato D, Falchook G, Zinner R, Wheler JJ, Janku F (2015). MET abnormalities in patients with genitourinary malignancies and outcomes with c-MET inhibitors. Clin Genitourin Cancer.

[30] Tang C, Fontes Jardim DL, Falchook GS, Hess K, Fu S, Wheler JJ (2013). MET nucleotide variations and amplification in advanced ovarian cancer: characteristics and outcomes with c-Met inhibitors. Oncoscience.

[31] Jardim DLF, Gagliato D de M, Falchook GS, Janku F, Zinner R, Wheler JJ (2014). MET aberrations and c-MET inhibitors in patients with gastric and esophageal cancers in a phase I unit. Oncotarget.

