# Supplementary material for: MET alterations detected in blood-derived circulating tumor DNA correlate with bone metastases and poor prognosis
Source: J Hematol Oncol. 2018 Jun 4;11:76. doi: 10.1186/s13045-018-0610-8 (PMC5987577; doi:10.1186/s13045-018-0610-8)
Supplement: Supplementary file 1 — Table S1. 54-gene panel (N = 122 patients)— identifies potential tumor-related genomic alterations within 54 cancer-related genes including amplifications in ERBB2, EGFR, and MET. Only non-synonymous alterations were analyzed. Table S2. 68-gene panel (N = 272 patients), comprising amplifications in 16 genes as well as some fusions and indels. Only non-synonymous alterations were analyzed. Table S3. 70-gene panel (N = 22 patients). Only non-synonymous alterations were analyzed. Table S4. Comparison of clinical characteristics in 438 patients with or without MET alterations (univariate analysis). (DOCX 20 kb) [file 13045_2018_610_MOESM1_ESM.docx]

**SUPPLEMENTAL MATERIAL**

**Supplemental Table 1. 54 gene panel (N = 122 patients):** identifies potential tumor-related genomic alterations within 54 cancer-related genes including amplifications in *ERBB2, EGFR*, and *MET.* Only non-synonymous alterations were analyzed.

| **GENES WITH COMPLETE EXON COVERAGE** | | | | | |
| --- | --- | --- | --- | --- | --- |
| *ALK* | *APC* | *AR* | *BRAF* | 18 GENES  **3 Copy Number**  **Variations in**  **BOLD** | |
| *CDKN2A* | ***EGFR*** | ***ERBB2*** | *FBXW7* |  |  |
| *KRAS* | ***MET*** | *MYC* | *NOTCH1* |  |  |
| *NRAS* | *PIK3CA* | *PTEN* | *PROC* |  |  |
| *RB1* | *TP53* |  |  |  |  |
|  | | | |  |  |
| **GENES WITH CRITICAL EXON COVERAGE** 36 GENES | | | | | |
| *ABL1* | *AKT1* | *ATM* | *CDH1* | *CSF1R* | *CTNNB1* |
| *ERBB4* | *EZH2* | *FGFR1* | *FGFR2* | *FGFR3* | *FLT3* |
| *GNA11* | *GNAQ* | *GNAS* | *HNF1A* | *HRAS* | *IDH1* |
| *IDH2* | *JAK2* | *JAK3* | *KDR* | *KIT* | *MLH1* |
| *MPL* | *NPM1* | *PDGFRA* | *PTPN11* | *RET* | *SMAD4* |
| ***SMARCB1*** | ***SMO*** | ***SRC*** | ***STK11*** | ***TERT*** | ***VHL*** |

**Supplemental Table 2. 68 gene panel (N = 272 patients)**, comprising amplifications in 16 genes as well as some fusions and indels. Only non-synonymous alterations were analyzed.

| **POINT MUTATIONS** (68 genes) | | | | **AMPLIFI-CATIONS** (16 genes) | **FUSIONS** (4 genes) | **INDELS** (1 gene) |
| --- | --- | --- | --- | --- | --- | --- |
| *AKT1* | *ALK* | ***APC*** | ***AR*** | *AR* | *ALK* | *EGFR* exon 19 deletions |
| *AFAR* | ***ARID1A*** | *ATM* | ***BRAF*** | *BRAF* | *RET* | *EGFR* exon 20 insertions |
| ***BRCA1*** | ***BRCA2*** | ***CCDN1*** | ***CCDN2*** | *CCNE1* | *ROS1* |  |
| ***CCNE1*** | *CDH1* | ***CDK4*** | ***CDK6*** | *CDK4* | *NTRK1* |  |
| ***CDKN2A*** | ***CDKN2B*** | *CTNNB1* | ***EGFR*** | *CDK6* |  |  |
| ***ERBB2*** | *ESR1* | *EZH2* | *FBXW7* | *EGFR* |  |  |
| ***FGFR1*** | ***FGFR2*** | *FGFR3* | *GATA3* | *ERBB2* |  |  |
| *GNA11* | *GNAQ* | *GNAS* | *HNF1A* | *FGFR1* |  |  |
| ***HRAS*** | *IDH1* | *IDH2* | *JAK2* | *FGFR2* |  |  |
| *JAK3* | ***KIT*** | ***KRAS*** | *MAP2K1* | *KIT* |  |  |
| *MAP2K2* | ***MET*** | *MLH1* | *MPL* | *KRAS* |  |  |
| ***MYC*** | ***NF1*** | *NFE2L2* | *NOTCH1* | *MET* |  |  |
| *NPM1* | ***NRAS*** | *NTRK1* | ***PDGFRA*** | *MYC* |  |  |
| ***PIK3CA*** | ***PTEN*** | *PTPN11* | ***RAF1*** | *PDGFRA* |  |  |
| *RET* | *RHEB* | *RHOA* | *RIT1* | *PIK3CA* |  |  |
| *ROS1* | *SMAD4* | *SMO* | *SRC* | *RAF1* |  |  |
| *STK11* | *TERT* | ***TP53*** | *VHL* | *Complete exon coverage for genes in **bold** | | |

**Supplemental Table 3: 70 gene panel (N = 22 patients)**

Only non-synonymous alterations were analyzed.

| **Complete Exon Sequencing** | | | | | | | | | |
| --- | --- | --- | --- | --- | --- | --- | --- | --- | --- |
| **Point Mutations (SNVs)**  (70 Genes) | | | | | | **Amplifications (CNVs)**  (18 Genes) | | **Fusions**  (6 Genes) | **Indels**  (3 Genes) |
| *AKT1* | *ALK* | *APC* | *AR* | *ARAF* | *ARID1A* | *AR* | *BRAF* | *ALK* | *EGFR** |
| *ATM* | *BRAF* | *BRCA1* | *BRCA2* | *CCND1* | *CCND2* | *CCND1* | *CCND2* | *FGFR2* | *ERBB2** |
| *CCNE1* | *CDH1* | *CDK4* | *CDK6* | *CDKN2A* | *CDKN2B* | *CCNE1* | *CDK4* | *FGFR3* | *MET*** |
| *CTNNB1* | *EGFR* | *ERBB2* | *ESR1* | *EZH2* | *FBXW7* | *CDK6* | *EGFR* | *NTRK1* |  |
| *FGFR1* | *FGFR2* | *FGFR3* | *GATA3* | *GNA11* | *GNAQ* | *ERBB2* | *FGFR1* | *RET* |  |
| *GNAS* | *HNF1A* | *HRAS* | *IDH1* | *IDH2* | *JAK2* | *FGFR2* | *KIT* | *ROS1* |  |
| *JAK3* | *KIT* | *KRAS* | *MAP2K1* | *MAP2K2* | *MET* | *KRAS* | *MET* |  |  |
| *MLH1* | *MPL* | *MYC* | *NF1* | *NFE2L2* | *NOTCH1* | *MYC* | *PDGFRA* |  |  |
| *NPM1* | *NRAS* | *NTRK1* | *PDGFRA* | *PIK3CA* | *PTEN* | *PIK3CA* | *RAF1* |  |  |
| *PTPN11* | *RAF1* | *RB1* | *RET* | *RHEB* | *RHOA* |  |  |  | **exons 19 & 20*  ***exon 14 skipping* |
| *RIT1* | *ROS1* | *SMAD4* | *SMO* | *SRC* | *STK11* |  |  |  |  |
| *TERT* | *TP53* | *TSC1* | *VHL* |  |  |  |  |  |  |

**Genes included on all three panel versions:** point mutations (SNVs) in *AKT1, ALK, APC, AR, ATM, BRAF, CDH1, CDKN2A, CTNNB1, EGFR, ERBB2, EZH2, FBXW7, FGFR1, FGFR2, FGFR3, GNA11, GNAQ, GNAS, HNF1A, HRAS, IDH1, IDH2, JAK2, JAK3, KIT, KRAS, MET, MLH1, MPL, MYC, NOTCH1, NPM1, NRAS, PDGFRA, PIK3CA, PTEN, PTPN11, RB1, RET, SMAD4, SMO, SRC, STK11, TERT, TP53, and VHL;* gene amplifications in *EGFR, ERBB2 (HER2), and MET.* Fusions and indel mutations were assessed for only in the 48 patients evaluated by the 68-gene and 70-gene panel.

**Supplemental Table 4. Comparison of clinical characteristics in 438 patients with or without *MET* alterations (univariate analysis).**

| Coexisting genetic alterations^1^ | Total patients, N=438 | *MET* wild- type, N=407 | *MET* altered, N=31 | P-value |
| --- | --- | --- | --- | --- |
| *TP53* | 149 (34.0%) | 129 (31.7%) | 20 (64.5%) | **4.7E-4** |
| *EGFR* | 53 (12.1%) | 43 (10.6%) | 10 (32.4%) | **0.001** |
| *PIK3CA* | 42 (9.6%) | 35 (8.6%) | 7 (22.6%) | **0.015** |
| *BRAF* | 27 (6.2%) | 22 (5.4%) | 5 (16.1%) | **0.023** |
| *ARID1A* | 19 (4.3%) | 15 (3.7%) | 4 (12.9%) | **0.023** |
| *ERBB2* | 24 (5.5%) | 20 (4.9%) | 4 (12.9%) | 0.071 |
| *ALK* | 14 (3.2%) | 11 (2.7%) | 3 (9.7%) | **0.047** |
| *KRAS* | 50 (11.4%) | 47 (11.5%) | 3 (9.7%) | 0.753 |
| *PTEN* | 9 (2.1%) | 6 (1.5%) | 3 (9.7%) | **0.007** |
| *ATM* | 13 (3.0%) | 11 (2.7%) | 2 (6.5%) | 0.251 |
| *SMAD4* | 12 (2.7%) | 10 (2.5%) | 2 (6.5%) | 0.207 |
| *KIT* | 12 (2.7%) | 10 (2.5%) | 2 (6.5%) | 0.207 |
| *MYC* | 20 (4.6%) | 18 (4.4%) | 2 (6.5%) | 0.604 |
| *NF1* | 19 (4.3%) | 17 (4.2%) | 2 (6.5%) | 0.552 |
| Metastatic site^2^ |  |  |  |  |
| Lymph nodes  Bone  Liver  Lung  Brain  Peritoneal  Adrenal  No metastases | 139 (31.7%)  102 (23.3%)  111 (25.3%)  90 (20.5%)  63 (14.4%)  49 (11.2%)  29 (6.6%)  147 (33.6%) | 123 (30.2%)  88 (21.6%)  101 (24.8%)  80 (19.7%)  56 (13.8%)  43 (10.6%)  25 (6.1%)  145 (35.6%) | 16 (51.6%)  14 (45.2%)  10 (32.3%)  10 (32.3%)  7 (22.6%)  6 (19.4%)  4 (12.9%)  2 (6.5%) | **0.016**  **0.004**  0.361  0.099  0.183  0.142  0.155  **0.005*** |
| ^1^genes altered in at least 2 patients with *MET* alterations have been included. ^2^Patients may have multiple sites of metastasis; only the sites with at least 2 patients with MET alterations have been included.  * “No metastases” was more common in patients with normal *MET.* | | | | |
